# Genetic variation and selection in the major histocompatibility complex Class II gene in the Guizhou pony

**DOI:** 10.7717/peerj.9889

**Published:** 2020-09-18

**Authors:** Chang Liu, Hongmei Lei, Xueqin Ran, Jiafu Wang

**Affiliations:** 1 College of Animal Sciences, Guizhou University, Guiyang, China; 2 College of Pharmacy, Guizhou University of Traditional Chinese Medicine, Guiyang, China; 3 Tongren University, Tongren, China

**Keywords:** Guizhou pony, MHC, Antigen binding sites, Evolution process, Adaptation

## Abstract

The Guizhou pony (GZP) is an indigenous species of equid found in the mountains of the Guizhou province in southwest China. We selected four regions of the equine leukocyte antigen (ELA), including *DQA*, *DRA*, *DQB,* and *DRB,* and used them to assess the diversity of the major histocompatibility complex (MHC) class II gene using direct sequencing technology. *DRA* had the lowest *d*_N_/*d*_S_ ratio (0.560) compared with the other three loci, indicating that *DRA* was conserved and could be conserved after undergoing selective processes. Nine *DQA*, five *DQB*, nine *DRA,* and seven *DRB* codons were under significant positive selection at the antigen binding sites (ABS), suggesting that the selected residues in ABS may play a significant role in the innate immune system of the GZP. Two GZP alleles were shared with Przewalski’s horse, and six older GZP haplotypes had a better relationship with other horse species by one or two mutational steps, indicating that the GZP may be a natural ancient variety of equid. The specific diversity of ABS and the numbers of unique haplotypes in the evolutionary process affords this species a better genetic fitness and ability to adapt to the native environment.

## Introduction

The major histocompatibility complex (MHC) genes play a major role in vertebrate immune systems and have a high degree of genetic diversity associated with the adaptive immune response and evolution (Lian et al., 2017; Kamath & Getz, 2011). The MHC system is divided into class I and class II, which are key parts of the immune system (Hughes & Nei, 1988). The MHC class II genes are highly polymorphic parts of the immune response that act by presenting extracellular antigens to T lymphocytes. These molecules are heterodimers with *α* and *β* chains encoded by A and B genes. The polymorphic sites of the class II genes are typically located at exon 2, which codes for the first extracellular domain or the antigen binding sites (ABS). The exon 2 codes for a section of the pocket of the MHC molecule. The ABS mainly encoded the second exon of the MHC class II gene and have more variation than the neighboring regions in this sequence (Li et al., 2014), indicating that ABS variation may help to determine the rates of evolution across the MHC (Hughes & Hughes, 1995). Previous studies have shown that exon 2 of MHC class II genes had the most polymorphisms and encoded the *α* and *β* domains principally responsible for peptide binding (O’Connor et al., 2007). The polymorphism of the MHC loci is commonly associated with different susceptibilities to infectious diseases (Hill, 2001), especially in sheep (Paterson, Wilson & Pemberton, 1998), mice (Meyer-Lucht & Sommer, 2005), voles (Kloch et al., 2010) and lemurs (Schad, Ganzhorn & Sommer, 2005). The equine MHC class II loci may assist in determining the host response to pathogens encountered by the horse (Miller et al., 2017). MHC variants play key roles in mate preference, kin recognition, and maternal-fetal interactions (Edwards & Hedrick, 1988; Bernatchez & Landry, 2003; Piertney & Oliver, 2006). The diverse functions and characteristics of MHC molecules is reflected in the evolutionary and adaptive processes within and between populations (Sommer, 2005).

The mechanisms of negative frequency-dependent selection (NFDS) and over-dominant selection have been well-studied in MHC genes. NFDS maintains intraspecific diversity and may interact with population density (Levitan & Ferrell, 2006; Meyer & Kassen, 2007). Over-dominant selection can maintain genetic polymorphisms in populations (Takahata & Nei, 1990). Correlative and experimental support for the negative frequency-dependent selection of MHC genes has been shown in humans (Trachtenberg et al., 2003), reed warblers (Westerdahl et al., 2004), mice (Kubinak et al., 2012), sticklebacks (Eizaguirre et al., 2012; Bolnick & Stutz, 2017) and guppies (Phillips et al., 2018). There are a number of examples of asymmetric over-dominant selection in populations found in the wild and in the laboratory (Landry & Bernatchez, 2001; Richman, Herrera & Nash, 2001; Lenz et al., 2009; Schwensow et al., 2010; Lenz et al., 2013). These results are supported by several computer-based binding prediction studies (Lenz, 2011; Lau et al., 2015; Buhler, Nunes & Sanchez-Mazas, 2016; Pierini & Lenz, 2018). Three primary sources of evidence currently support the idea of balancing selection: (i) elevated levels of polymorphisms, (ii) the rates of nonsynonymous (*d*_N_) to synonymous (*d*_S_) nucleotide substitutions (Hughes & Nei, 1988; Hughes & Nei, 1989), and (iii) trans-species polymorphisms with alleles among species (Klein et al., 1993). The *d*_N_/*d*_S_ ratio is frequently used to measure selective pressure on genes (Yang et al., 2000), and more specifically, the markedly different rates of evolution across the MHC genes (Hughes & Hughes, 1995). Site-specific methods have found elevated *d*_N_/*d*_S_ ratios at ABS, suggesting substantially different rates of evolution across the MHC. MHC variation within species and among species has proven to be useful in determining the historical patterns of selection in various mammals (Cutrera & Lacey, 2007).

In the family Equidae, the horse MHC class II gene, also known as equine leukocyte antigen (ELA) class II, is located on the short arm of chromosome 20q14-q22 (Mäkinen et al., 1989; Ansari et al., 1988). It contains the *DQA*, *DQB*, *DRA,* and *DRB* genes. The *DQA* and *DRA* genes encode for the *α*-chain of ELA class II molecules, and the polymorphisms of the *DQA* and *DRA* genes have been determined in European equids (Luís et al., 2005; Janova et al., 2009; Kamath & Getz, 2011). The *DQB* and *DRB* genes encode the *β*-chain of the ELA class II complex, and high levels of *DRB* and *DQB* polymorphisms have been reported in Arabian and European horses (Fraser & Bailey, 1996; Mashima, 2003). Previous reports indicated that exon 2 of the ELA class II gene is genetically diverse among horse populations (Kamath & Getz, 2011). We examined the sequence variation in the second exon to determine the selective pressures and evolutionary path for the Guizhou pony.

The Guizhou pony is an indigenous species that was found in the Guizhou province during the Warring States Period (475-221 B.C.) in Ancient China. It is one of five Chinese pony species and has a body height of only 1.1 m (10-11 hands). The mtDNA/SSR polymorphism has been determined in several pony populations derived from native Irish, Canadian, and Chinese breeds using mtDNA/SSR markers (McGahern et al., 2006; Prystupa et al., 2012). We sought to analyze the variation in the MHC II exon 2 of the *DQA*, *DRA*, *DQB*, and *DRB* regions and their relationship with the selection and evolution in the GZP.

## Material and Methods

### Animal collection and DNA isolation

A total of 50 blood samples were collected from GZP in Ziyun County, Anshun, Guizhou Province, China. All ponies used in our study were 4 to 8 years old. All animal procedures were approved by the Institutional Animal Care and Use Committee of Guizhou University (Approval number EAE-GZU-2018-P007). The GZP were randomly selected and were all well-developed and in good health, with heights ranging from 102 to 118 cm and weights between 210 to 265 kg. Blood samples were collected from the jugular vein and were kept in EDTA Na2. All samples were stored at −20 °C until DNA extraction. Genomic DNA was extracted from blood samples using the SQ Blood DNA Kit (OMEGA, USA). The nucleic acid concentration of the extracted genomic DNA was calculated by determining OD260/OD280, and detected by 0.7% agarose gel electrophoresis.

**Table 1:** The primers for DRA, DRB, DQA and DQB gene detection.

Gene name	Primer name	Primer sequence (5′→3′)	Length (bp)
DRAexon2	DRA-F	AGGATCACGTGATCATCCAG	246
	DRA-R	CATTGGTGTTTGGAGTGTTG	
DRBexon2	DRB-F	CTCTGCAGCACATTTCCTGGAG	276
	DRB-R	CGCCGCTGCACCAGGAA	
DQBexon2	DQB-F	CTCGGATCCGCATGTGCTACTTCACCAACG	230
	DQB-R	GAGCTGACGGTAGTTGTGTCTGCACAC	
DQAexon2	DQA-F	CTGATCACTTTGCCTCCTATG	246
	DQA-R	TGGTAGCAGCAGTAGAGTTG	

### PCR amplification, cloning, and sequencing

The exon 2 regions of the *ELA-DQA*, *DQB*, *DRA,* and *DRB* genes were amplified from genomic DNA using PCR with specific primers. We amplified 246 bp of the *DRA* using the equid-specific primers *DRA*-F and *DRA*-R (Albright-Fraser et al., 1996), 246 bp of the *DQA* using the primers *DQA*-F and *DQA*-R (Fraser & Bailey, 1998), 276 bp of the *DRB* using the primers *DRB*-F and *DRB-*R (Fraser & Bailey, 1996), and 230 bp of the *DQB* using the primers *DQB*-F and *DQB*-R (Mashima, 2003). All primers were synthesized by the Bio-Engineering Company (Shanghai, China) (Table 1). The total PCR volume was 20 µL, and contained 10 µL of 2 × PCR Mixture (0.1 U Taq Plus Polymerase/µL, 500 µM dNTP each, 20 mM Tris–HCl (pH8.3), 100 mM KCl, 3 mM MgCl_2_), 0.4 µL of upstream/downstream primers (10 µmol/L), and 1 µL templates. PCR amplification was carried out with initial denaturation at 95 °C for 5 min, followed by 30 cycles (95 °C for 30 s, 58 °C for 30 s, and 72 °C for 30 s), and a final extension at 72 °C for 10 min. PCR products were extracted and purified using the Gel Extraction Kit (OMEGA, USA), and were ligated into pGEM^®^-T vectors and transformed into *E. coli* competent cells. Twenty positive clones of each sample were removed with a sterile toothpick and were detected using the Sanger sequencing method (Invitrogen, China). Alleles were confirmed if the same allele was observed in at least two different individuals.

### DNA sequence polymorphism analysis

The base composition of the *DRA*, *DRB*, *DQA* and *DQB* genes was analyzed using MEGA7 software (Kumar, Stecher & Tamura, 2016). Standard descriptive diversity indices for each locus within the GZP were calculated using MEGA7 software, including the variable sites (V), parsim-info sites (P), singleton sites (S), and the transition/transversion bias ratio (R). It was important to ascertain whether the variability was uniformly distributed or was confined to small segments of the variable regions when determining the nature of the variable region. The Wu–Kabat variability index was calculated using the formula by Wu and Kabat Wu & Kabat (1970) with respect to amino acids at peptide-binding pockets. The variation of amino acids was calculated by the mutation rate (variability = number of different amino acids at a certain position/frequency of the most common amino acids at this position) (Wu & Kabat, 1970). Selection was estimated using MEGA7 software in terms of the relative rates of nonsynonymous (*d*_N_) and synonymous (*d*_S_) mutations, according to Nei and Gojobori’s method with the Jukes and Cantor (JC) correction (Nei & Gojobori, 1986). The selection *Z*-Test (*P* < 0.05) was performed for all sites under the null hypothesis of neutrality (*d*_N_ = *d*_*S*_) and the alternative hypotheses of non-neutrality (*d*_N_ ≠ *d*_S_), positive selection (*d*_N_ >  *d*_S_), and purifying selection (*d*_N_ <  *d*_S_).

### Site-specific selection analyses and protein 3D structure analysis

We estimated the nonsynonymous and synonymous substitutions in the overall domain, ABS, and non-ABS for the *DQA*, *DQB*, *DRA* and *DRB* alleles. We assessed the positive selection using CodeML subroutine in the PAML program (Yang, 2007), which was more sensitive than other methods for assessing selection at the molecular level (Anisimova, Bielawski & Yang, 2001). The PAML program used the maximum likelihood estimation to examine heterogeneity in rates of *ω* = *d*_N_/*d*_S_ among codons (Bielawski & Yang, 2003). The PAML program was able to better detect the molecular evidence of selection compared to other programs (Anisimova, Nielsen & Yang, 2003). We assessed heterogeneity in *ω* (*ω*  <  1: purifying selection, *ω* = 1: neutral evolution, *ω* >  1: positive selection) across the four alleles (*DQA*, *DQB*, *DRA* and *DRB*) to identify codons under positive selection. The observed *ω* value followed six models in PAML: M0 (one ratio, average *ω* across all sites), M1a (nearly neutral), M2a (positive selection), M3 (discrete), M7 (beta), and M8 (beta and omega) (Yang et al., 2000). We used the online SWISS-MODEL program (Biasini et al., 2014; Waterhouse et al., 2018) (https://swissmodel.expasy.org/interactive) to make predictions about the *DQA*, *DQB*, *DRA* and *DRB* protein structures.

### Phylogenetic allele networks

We constructed a median-joining haplotype network to infer the phylogenetic relationships among the sequence haplotypes (Bandelt, Forster & Röhl, 1999) using the maximum parsimony in Network 4.6.1 (http://www.fluxus-engineering.com/sharenet.htm). The haplotype median networks of *DQA*, *DQB*, *DRA* and *DRB* between GZP and known horse species (*Eqca*, *E.callabus*; *Eqpr, E.przewalski*; *Eqki*, *E.kiang*; *Eqgr*, *E.grevyi*; *Eqas*, *E.asinus*; *Eqbu*, *E.burchelli*; *Eqze, E.zebra*; *Eqhe*, *E.hemionus*) from GenBank were plotted using NetWork 4.6. The frequency information and population proportion of the alleles were incorporated into the visualization of the network. Sequences from the horse, including *E. callabus*, *E. przewalskii*, *E. burchellii*, *E. asinus*, were incorporated to evaluate the distance from the Guizhou pony’s haplotypes (Table 2).

## Results

### Analysis of nucleotide diversity

184 alleles were identified from 1,000 sequencing clones, with 118 effective alleles selected from the total. Of the 118 alleles, there were 18 novel *DQA* alleles (GenBank accession number: MT304744 –MT304761), 38 new *DQB* alleles (MT304705 –MT304743), 22 new *DRA* alleles (MT304762 –MT304783) and 28 new *DRB* alleles (MT304784 –MT304811) (Table S1). The alignment results are listed in Table S1 for the effective alleles from *DQA*, *DQB, DRA*, *DRB* and the sequences of JQ254059, AF034122, AJ575295, and AF144564. A considerable sequence diversity within the genus was revealed based on the *DQA*, *DQB*, *DRB* alignment results. The nucleotide diversity in *DRA* was much lower than in *DQA*/*B* and *DRB* in GZP, which is comparable with the level of nucleotide diversity in *DRA* from other species in the Equus genus (Kamath & Getz, 2011). Within the GZP, the genetic diversity was much higher in *DQA*, *DQB,* and *DRB* than in *DRA* and the ratio (variable sites/length) was the lowest at the *DRA* locus (15.04%) and highest at the DQB locus (46.08%).

### Analysis of nucleotide compositions

The GC contents of *DQB* and *DRB* were higher than those of *DQA* and *DRA* (Table S2). The content of G+C (48.10%) was slightly lower than that of A+T (51.90%) at *DQA*, and the content of G+C (48.10%) was lower than that of A+T (51.90%) at *DRA*, which revealed that *DQA* and *DRA* had lower GC percentages. However, the base composition of G+C (64.20%) was higher than that of A+T (35.80%) in *DQB* alleles, and the base composition of G+C (61.90%) was more than that of A+T (38.10%) in DRB alleles, revealing that *DQB* and *DRB* had a higher GC content. The R (transitions/transversions) was 1.357 and 2.241 in the *DQA* and *DRA* alleles, respectively. However, there was an R of 0.778 and 0.573 in the *DQB* and *DRB* alleles, respectively. Our results revealed that the *DRA* locus was more well-conserved than the other loci.

**Table 2:** General information regarding the horse populations analyzed in this Network study.

Locus	Breed	Source	GenBank ID
*DQA*	*Guizhou pony*	This study	MT304744 –MT304761
*DQA*	*Equus przewalskii*	NCBI	JX088699, JX088698, JX088697, JX088696, U92509, U92509
*DQA*	*Equus caballus*	NCBI	AF115329, AF115328, AF115327, AF115326, AF115325, AF115324, U92508, U92519, U92518, U92517, U92516, U92515, U92514, U92513, U92512, U92511, U92510, U92507, U92506, U92505
*DQA*	*Equus burchellii*	NCBI	EU935835, EU935837, EU935836, EU935834, EU935833, EU935832, EU935829, EU930130 HQ637409, HQ637408, HQ637407, HQ637397, HQ637406, HQ637405, HQ637404, HQ637403, HQ637402, HQ637401, HQ637400, HQ637399, HQ637398
*DQA*	*Equus zebra*	NCBI	EU935838, EU935831, EU935830, EU935828
*DQA*	*Equus grevyi*	NCBI	EU930136, EU930131
*DQA*	*Equus asinus*	NCBI	U92522, U92521
*DQA*	*Equus hemionus*	NCBI	U92520, EU930135
*DQA*	*Equus kiang*	NCBI	EU930134, EU930133, EU930132
*DQB*	*Guizhou pony*	This study	MT304705 –MT304743
*DQB*	*Equus asinus*	NCBI	AF034125, AF034124, AF034123, AF034122, U31776, U31775, U31774, XM_014839831.1
*DQB*	*Equus przewalskii*	NCBI	XM_008508365.1
*DQB*	*Equus caballus*	NCBI	JQ254069.1, L33910.1, XM_005603501.3, JQ254075.1, JQ254071.1, NM_001317256.1
*DRA*	*Guizhou pony*	This study	MT304762 –MT304783
*DRA*	*Equus hemionus*	NCBI	L47173, EU930128
*DRA*	*Equus kiang*	NCBI	FJ657514, EU930127
*DRA*	*Equus grevyi*	NCBI	EU930125, EU930116
*DRA*	*Equus zebra*	NCBI	EU930117, EU930119, EU930123, EU930124, EU930129
*DRA*	*Equus burchellii*	NCBI	EU930118, EU930120, EU930121, EU930122, EU930126, HQ637392, HQ637393, HQ637394, HQ637396, HQ637395, AJ575299
*DRA*	*Equus caballus*	NCBI	L47172, L47174, AJ575295, JN035631, JN035630, JN035629
*DRA*	*Equus asinus*		AJ575298, AJ575297, AJ575296, HM165492, FJ487912, L47171, AF541938
*DRB*	*Guizhou pony*	This study	MT304784 –MT304811
*DRB*	*Equus przewalskii*	NCBI	AF084188.1, XM_008511984.1
*DRB*	*Equus caballus*	NCBI	L76972, L76978, L76976, L76975, L76974, L76977, L76973, AF170067, L77079, AF144564, XM_023624024.1, JQ254087.1 XR_002801945.1, XR_001379170.2, XM_023624023.1, XM_014734203.2, XM_014734205.1, NM_001142816.1, JN035625.1, JQ254096.1, JQ254095.1, L25644.1, JN035627, JN035623.1, JN035622.1, JN035621.1, JN035624.1, JQ254099.1, JN035626.1, JQ254093.1
*DRB*	*Equus asinus*	NCBI	XM_014846373.1, XR_001398881.1, XM_014846372.1, KJ596517.1, KJ596507.1, KJ596519.1, KJ596510.1, KJ596510.1, KJ596516.1, KJ596511.1, KJ596518.1, KJ596512.1, KJ596514.1, KJ596515.1

### The amino acid composition analyses

**Figure 1:**
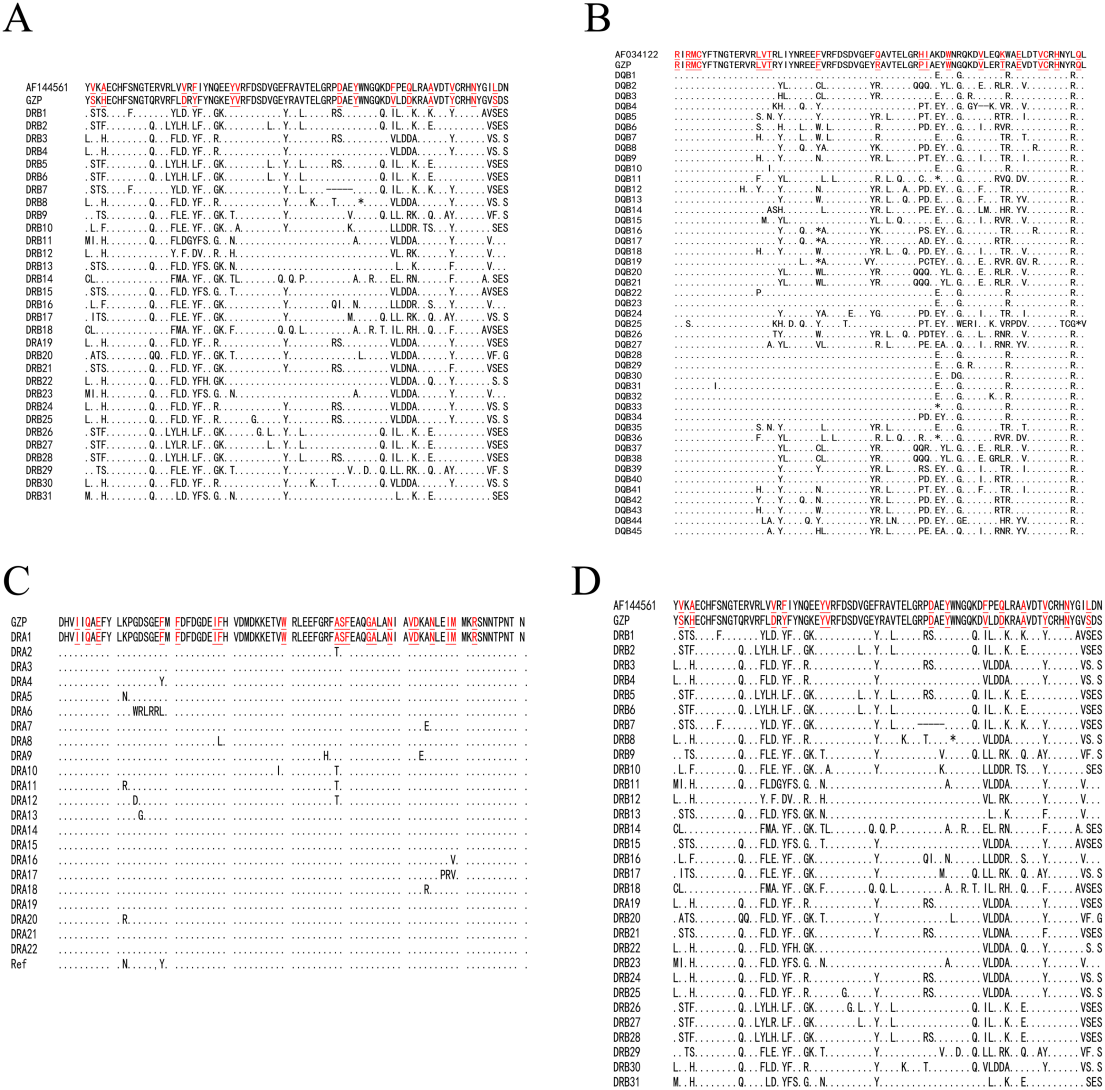
The amino acid alignment of the *DQA* (A), *DQB* (B), *DRA* (C) and *DRB* (D) locus. Underlines below amino acids indicated antigen binding sites (ABS). The missing amino acid was denoted with hyphen.

**Figure 2:**
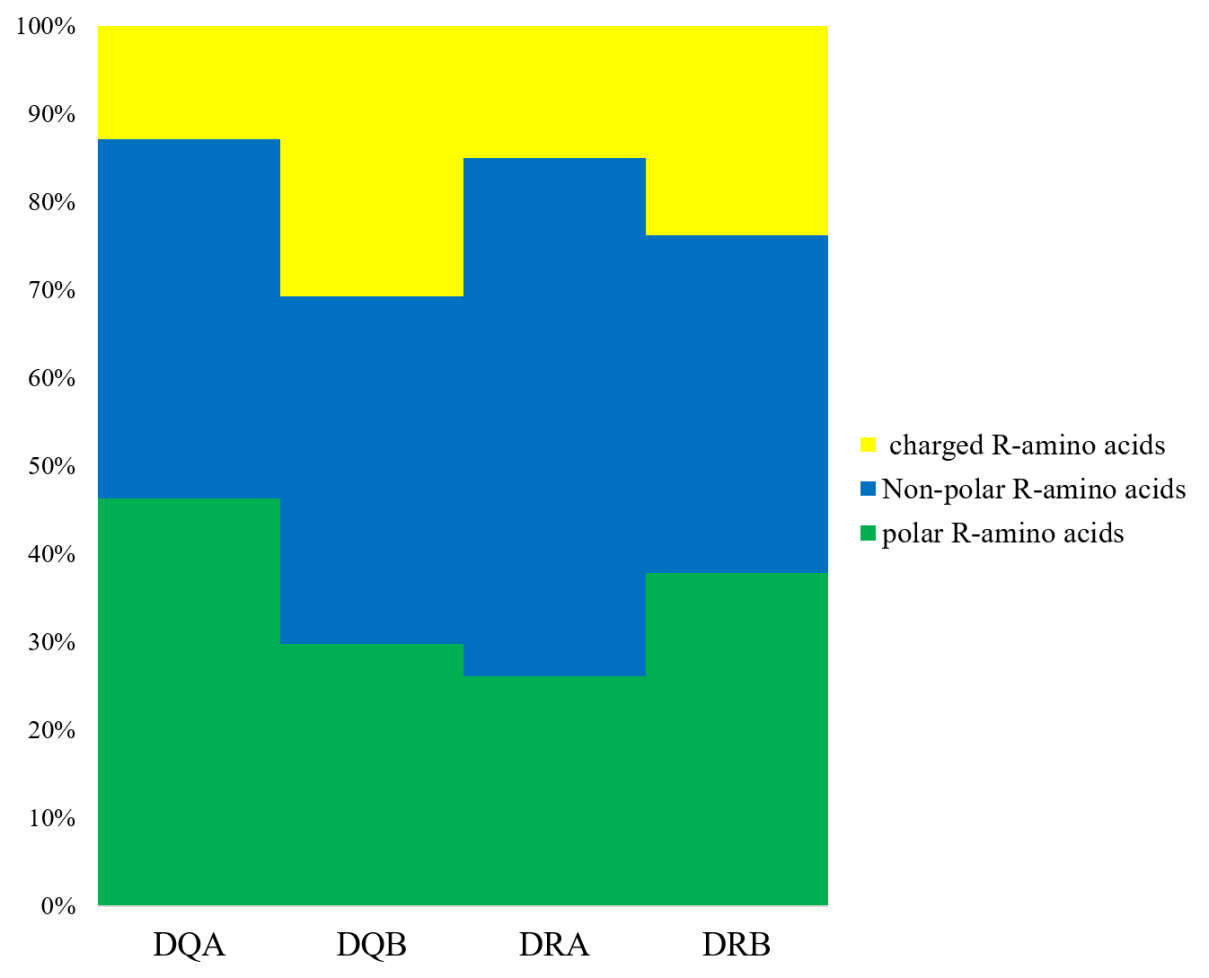
The distribution of amino acids with non-polar, polar and positively, negatively charged residues. Non-polar R-amino acids: Ala, Leu, Val, Trp, Ile, Phe, Pro, Met; polar R-amino acids: Gly, Cys, Ser, Tyr, Thr, Asn, Gln; charged R-amino acids: Arg, Lys, His, Glu, Asp.

We determined that the exon 2 of the *DQA*, *DQB*, *DRA,* and *DRB* nucleotide sequences encoded 82, 76, 81, and 79 amino acid sequences, respectively. The underlined residues in Fig. 1 indicated an assumed ABS, based on the HLA equivalents (Brown et al., 1988; Brown et al., 1993), and may contact the antigen peptides (Figs. 1A–1D). There were 38 (45.12%), 51 (67.10%), 16 (19.75%), and 49 sites (62.02%) that were variable in the predicted amino acid sites of the *DQA*, *DQB*, *DRA* and *DRB* of GZP populations, respectively. For the ABS, 17 of 21 sites (80.95%), 12 of 18 sites (66.67%), 5 of 20 sites (25.00%), and 13 of 14 sites (92.85%) were diverse at the *DQA*, *DQA*, *DQB*, *DRA* and *DRB* loci. The amino acid compositions of *DQA*, *DQB*, *DRA,* and *DRB* at ABS were calculated using MEGA 7 software (Fig. 2). There were more polar R-amino acids at the *DQA* locus (46.30%), and included Gly, Cys, Ser, Tyr, Thr, Asn, and Gln (Fig. 2). The non-polar R-amino acids at the *DRA* locus had the highest percentage (58.86%), and included Ala, Leu, Val, Trp, Ile, Phe, Pro, and Met (Fig. 2). The largest proportion of charged R-amino acids (30.70%) was located at *DQB*, and consisted of Arg, Lys, His, Glu, and Asp (Fig. 2).

### Global selection analyses

**Figure 3:**
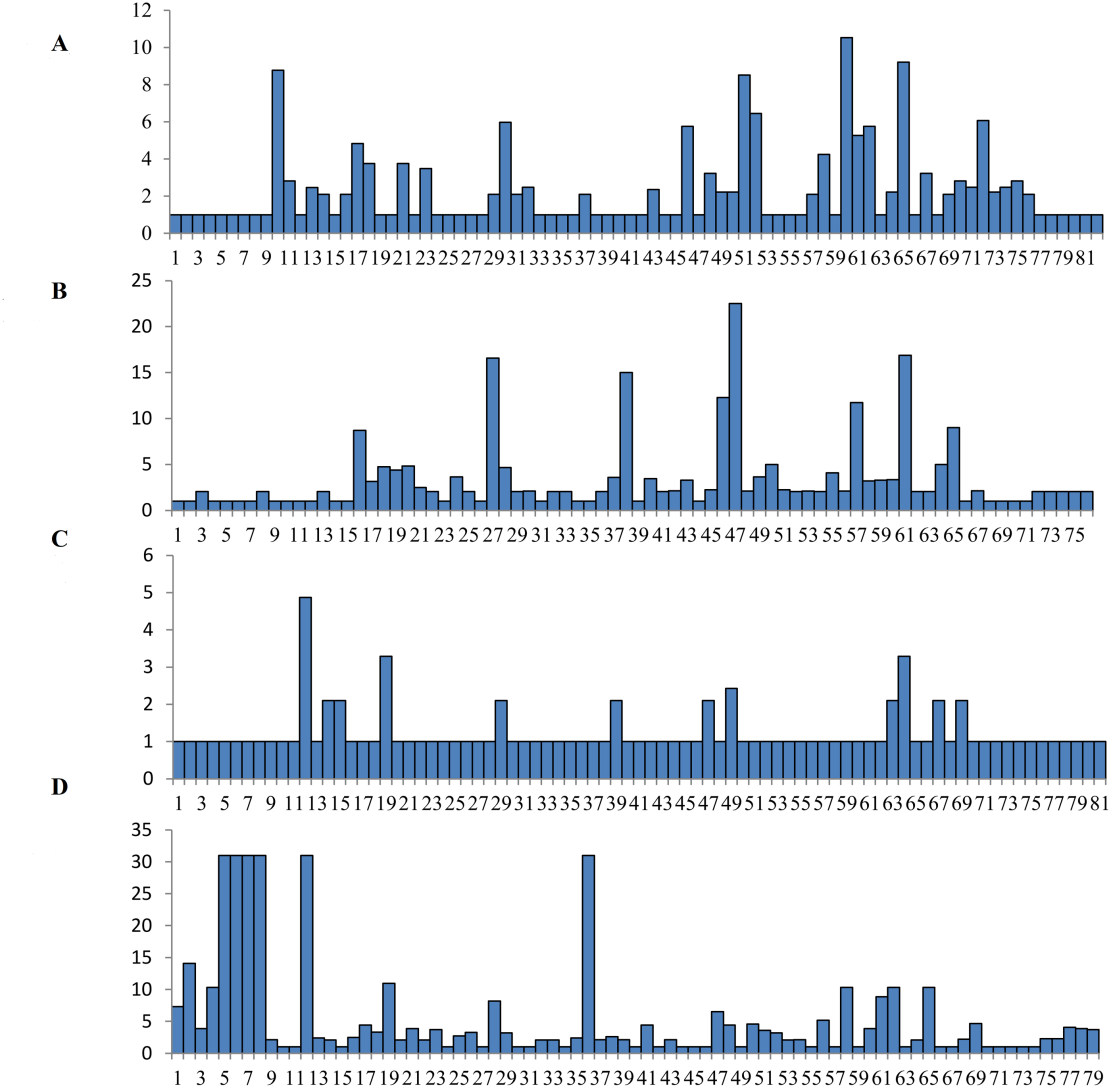
Wu-Kabat variability index of *DQA* (A), *DQB* (B), *DRA* (C) and *DRB* (D) loci.. The vertical index indicated the Wu-Kabat index at each amino acid position. The horizontal axis showed the consensus amino acids in the DQA, DQB, DRA and DRB peptides.

The Wu-Kabat variability index was not used to select all of the variable amino acids (Wu & Kabat, 1970). A total of fifteen amino acids at the *DQA* locus were strongly selected at residues 10, 17, 18, 21, 23, 30, 46, 51, 52, 58, 60, 61, 62, 65 and 72, with the highest variability occurring at residue 60 (Fig. 3). Many polymorphic sites were observed at the *DQB* locus, with eight high mutation loci at residues at 16, 27, 38, 46, 47, 57, 61, and 65 (Fig. 3). 12 residues were found to be polymorphic at the DRA locus, including residues at 12, 14, 15, 19, 29, 39, 47, 49, 63, 64, 67 and 69 (Fig. 3). Amino acid residues at six different positions in the *DRB* locus had high values (more than 30) on the Wu-Kabat variability index, and the strongly selected amino acid regions were found at residues 1, 2, 4, 5, 6, 7, 8, 12, 19, 28, 36, 47, 48, 50, 56, 58, 61, 62, 65 and 69 (Fig. 3). A comparison of the *d*_N_/*d*_S_ ratio averaged across the whole coding region suggested that positive selection occurred at loci *DQB* (*d*_N_/ *d*_S_ = 1.127, *p* = 0.322) and *DRB* (*d*_N_/*d*_S_ = 1.228, *p* = 0.202), and purifying selection appeared at *DQA* (*d*_N_/*d*_S_ = 0.779, *p* = 0.143) and *DRA* (*d*_N_/*d*_S_ = 0.560, *p* = 0.069) (Table 3). All codon sites were not statistically significant according to the *Z*-tests (*p* > 0.05, Table 3). The estimates of *d*_N_/*d*_S_ suggested that *DQA* and *DRA* were not affected by the positive selection at the genetic level.

### Site-specific selection analyses

It is unlikely for selection to act uniformly across MHC genes over evolutionary time. Selection was more likely to occur at specific codons based on their functional role. The rate of nonsynonymous substitutions for the ABS (*d*_N_ = 0.594 ± 0.132) exceeded the number of synonymous substitutions four times (*d*_S_ = 0.128 ±  0.080) at the *DRB* (Table 3). Our results are in agreement with those observed in the Argentine Creole horse, which exhibited rates of nonsynonymous substitutions more than four times the number of synonymous substitutions at exon 2 of *ELA-DRB* (Díaz et al., 2001). The ABS rates of synonymous substitutions and nonsynonymous substitutions for *DQA* and *DQB* were similar (*d*_N_ = 0.330 ± 0.088, *d*_S_ = 0.287 ± 0.110; *d*_N_ = 0.206 ± 0.079, *d*_S_ = 0.133 ±  0.076, respectively) (Table 3). The ABS sites at the *DRA* exhibited less nonsynonymous substitutions (*d*_N_ = 0.017 ±  0.008) than synonymous substitutions (*d*_S_ = 0.028 ± 0.022) with a *d*_N_/*d*_S_ ratio of 0.607 (Table 3). *Z*-tests performed separately on ABS were significant for *DRB* (*p* = 0.001) providing evidence for positive selection at these sites. We could not reject the null hypothesis of neutral evolution at the non-ABS site (Table 3). The *Z*-tests by site type at the *DQA*, *DQB,* and *DRA* sites could not reject the null hypothesis of neutrality (*p* > 0.05). In contrast, the non-ABS sites showed more synonymous substitutions than nonsynonymous substitutions with *d*_N_/*d*_S_ ratios of 0.581, 0.895, 0.520, and 0.678 at *DQA*, *DQB*, *DRA* and *DRB,* respectively (Table 3).

**Table 3:** The Indices of selection at the *DQA*, *DQB*, *DRA* and *DRB* loci.

Allele	Type	No.	aa distance	*d*_N_	*d*_S_	*d*_N_/ *d*_S_	Z; *d*_N_≠*d*_S_	Z; *d*_N_ > *d*_S_	Z; *d*_N_ < *d*_S_
*DQA*	All	82	0.213 ± 0.029	0.138 ± 0.024	0.177 ± 0.042	0.779	0.296	1.000	0.143
	ABS	21	0.416 ± 0.066	0.330 ± 0.088	0.287 ± 0.110	1.150	0.728	0.361	1.000
	non-ABS	61	0.148 ± 0.031	0.086 ± 0.021	0.148 ± 0.041	0.581	0.123	1.000	0.061
*DQB*	All	76	0.155 ± 0.026	0.097 ± 0.018	0.086 ± 0.018	1.127	0.621	0.322	1.000
	ABS	19	0.264 ± 0.071	0.206 ± 0.079	0.133 ± 0.076	1.549	0.248	0.123	1.000
	non-ABS	57	0.124 ± 0.024	0.068 ± 0.014	0.076 ± 0019	0.895	0.734	1.000	0.371
*DRA*	All	81	0.028 ± 0.007	0.014 ± 0.004	0.025 ± 0.007	0.560	0.138	1.000	0.069
	ABS	20	0.042 ± 0.019	0.017 ± 0.008	0.028 ± 0.022	0.607	0.649	1.000	0.317
	non-ABS	61	0.023 ± 0.007	0.013 ± 0.004	0.025 ± 0.008	0.520	0.133	1.000	0.077
*DRB*	All	79	0.212 ± 0.028	0.141 ± 0.023	0.114 ± 0.025	1.228	0.429	0.202	1.000
	ABS	15	0.524 ± 0.057	0.594 ± 0.132	0.128 ± 0.080	4.640	0.001	0.001	1.000
	non-ABS	64	0.144 ± 0.025	0.076 ± 0.015	0.112 ± 0.029	0.678	0.247	1.000	0.129

**Notes.**

aa distance average pair-wise amino acid distance;

*d*_N:_ nonsynonymous, *d*_S:_ synonymous, *Z* test *p*-values for rejecting the null hypothesis of neutrality (*d*_N_ = *d*_S_) for the alternative hypotheses of non-neutrality (*d*_N_ ≠ *d*_S_), positive selection (*d*_N_ >  *d*_S_), and purifying selection (*d*_N_ < *d*_S_).

**Figure 4:**
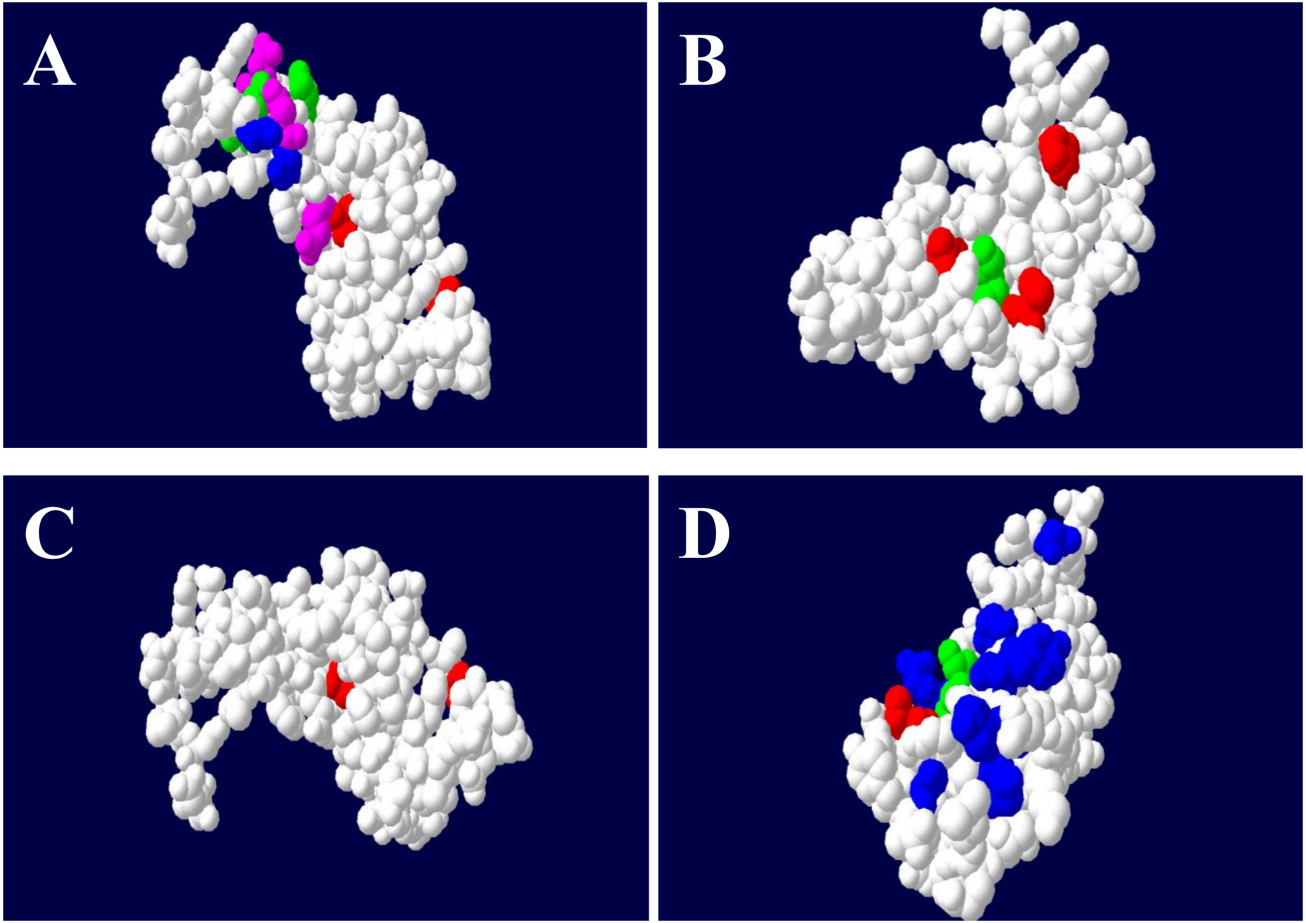
The ABS binding residues of *DQA* (A), *DQB* (B), *DRA* (C) and *DRB* (D) in GZP. The non-ABS region was circled in white color, the equivalent position of ABS were in red (Non-polar R-amino acids), blue (polar R-amino acids), green (positively charged R-amino acids) and purple (negatively charged R-amino acids), respectively.

The results from the selection analyses in PAML revealed different levels of selection for the four loci (Table 4). The variable evolutionary rates across the codon sites (M3) fit our data better than the M0 model and models M2a and M8 had higher log-likelihoods than positive selection (M1a and M7). The M2a and M8 models implied that approximately 2% of sites may be under positive selection at the *DQA* site (*ω* = 8.583, *ω* = 8.425) (Table 4). The posterior means of *ω* were estimated across the *DQA* codons under positive selection models and predicted fourteen sites (positions 10, 17, 18, 30, 46, 51, 52, 60, 61, 65, 68, 69, 70, 72) that may be under selection (*ω* > 1), nine (10, 30, 46, 51, 52, 61, 65, 68, and 72) of which were also putative ABS, based on the HLA equivalents (Fig. 4). However, the discrete model (M3: 3 discrete evolutionary rate classes) had the highest log-likelihood and estimated that only 6.6% of codon sites had *ω* values greater than one (*ω* = 10.031) with the remaining 93.4% of sites being assigned *ω* values close to 0 (Table 4).

However, the posterior means of *ω* across *DQB* codon sites estimated by M2a (*ω* = 6.240) and M8 (*ω* = 6.373) predicted that only five codons were under significant positive selection (positions 16, 27, 47, 57, 61). These five codons were also known as putative ABS based on the HLA equivalents (Fig. 4). The M3 model at the *DQB* estimated that approximately 8% of codon sites had *ω* values greater than one (7.3% with *ω* = 1.283; 0.6% with *ω* = 6.935) with the remaining 92% of sites being assigned *ω* values close to 0 (*ω* = 0.054) (Table 4).

The M3 model estimated that only 6.6% of codon sites had *ω* values greater than one (*ω* = 10.031) with the remaining 93.4% of sites being assigned *ω* values close to 0 for the *DRA* (Table 4). Moreover, the posterior means of *ω* across the *DRA* codon sites estimated by M2a (*ω* = 10.286) and M8 (*ω* = 10.323) predicted that nine codons (positions 12, 14, 15, 16, 18, 19, 49, 64, 68) were under significant positive selection. However, only two sites (19 and 49) were known as putative ABS based on the HLA equivalents (Fig. 4).

The M3 model estimated that 14.6% of codon sites had *ω* values greater than one (*ω*1 = 1.351, *ω*2 = 6.823) at the *DRB* (Table 4), which was higher than the other MHC class II codons (*DQA*, *DQB* and *DRA)*. The posterior means of *ω* across the *DRB* codon sites were estimated by the M2a (*ω* = 5.972) and M8 (*ω* = 5.961) models and predicted that twelve codons (positions 1, 2, 23, 28, 47, 48, 58, 61, 62, 65, 69, and 77) were under significant positive selection, seven (2, 28, 48, 58, 61, 69, and 77) of which were also putative ABS based on the HLA equivalents (Fig. 4).

### Evolutionary analysis

**Figure 5:**
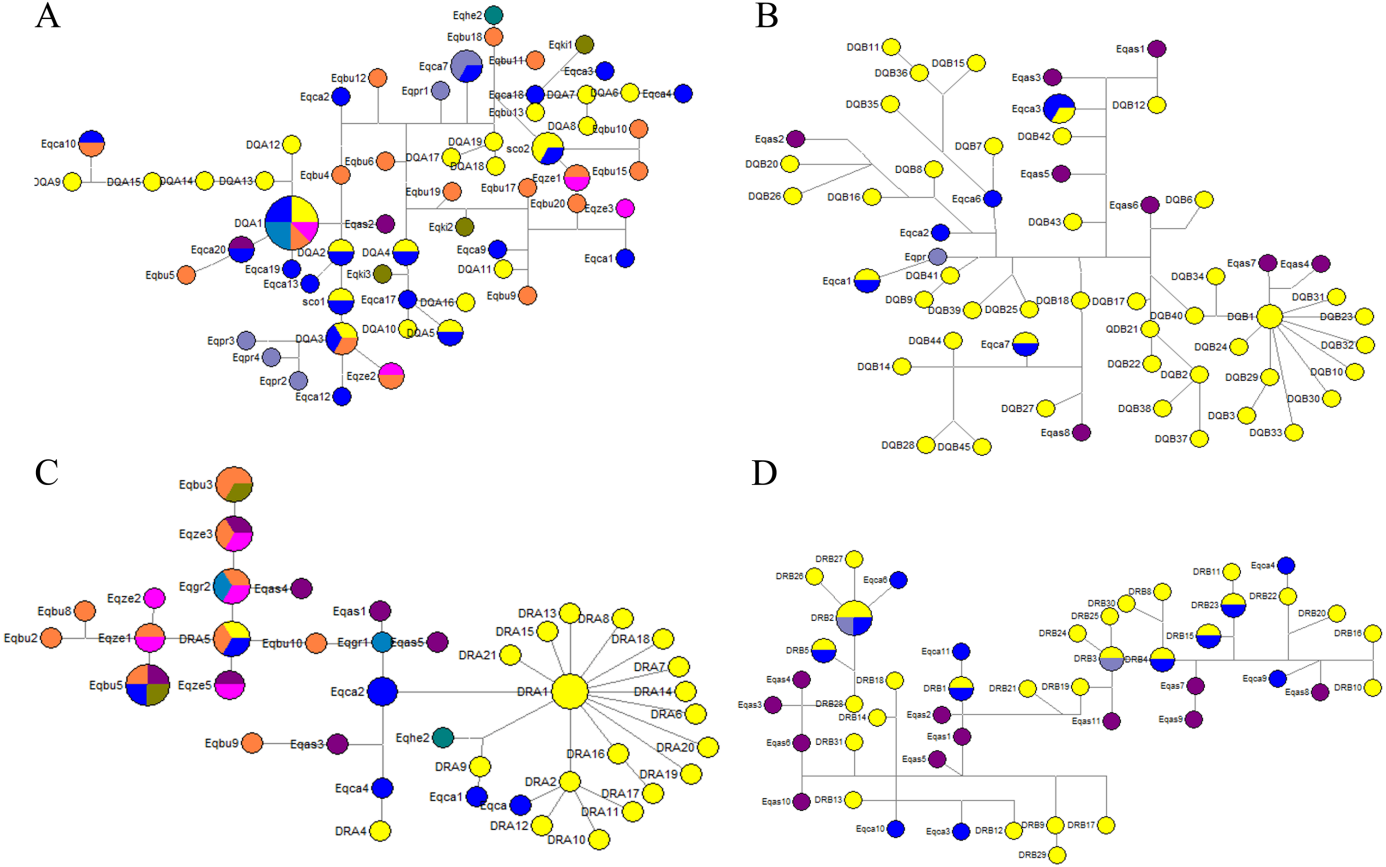
Median-joining network of DQA (A), DQB (B), *DRA (C) and DRB (D)* sequences in the Equidae family. The circle size was proportional to haplotype frequency.

We could not determine the genealogy of *DQA*, *DQB*, *DRA* and *DRB* due to the presence of loops in the network (Figs. 5A–5D). Some alleles were more likely to be ancestral based on their internal position in the network and a greater frequency of mutational connections. These alleles seemed more likely to be ancestral at the *DQA* locus, including *DQA1*, *DQA3*, *Eqca17,* and *Eqca18*. Allele *DQA1* appears to be ancestral for most alleles, namely *DQA12*, *DQA13*, *DQA14*, *DQA15*, *DQA9*, *Eqca10*, *Eqbu6*, *Eqca20*, *Eqas1*, *Eqas2*, *Eqbu5*, *Eqca19*, *Eqbu4*, and *Eqbu12*. Three haplotypes of Przewalski’s horse *(Eqpr3*, *Eqpr4* and *Eqpr2*) were separated from *DQA3* by two mutational steps and were most closely related to GZP haplotypes. Meanwhile, the *DQA1* allele was shared among four species (*Eqca15*, *Eqgr1*, *Eqbu2* and *Eqze1*), *DQA2* was shared with *Eqca14*, and *DQA3* was shared between two species (*Eqca16* and *Eqbu20*). At the *DQB* locus, allele *DQB1* appears to be ancestral for most alleles, including *DQB31*, *DQB23*, *DQB32*, *DQB10*, *DQB30*, *DQB33*, *DQB29*, *DQB3*, *DQB24*, *DQB34*, *DQB40*, *Eqas7* and *Eqas4*. We found that haplotypes *DQB5* and *DQB13* were shared between *Eqca1* and *Eqca7*, respectively. Allele *DRA5* was shared between *Eqbu7* and *Eqca5*, and *DRA1* was shared with *DRA3* for the *DRA* locus. Interestingly, allele *DRA1* seemed more likely to be ancestral, containing twenty alleles, including *DRA21*, *DRA15*, *DRA13*, *DRA8*, *DRA18*, *DRA7*, *DRA14*, *DRA6*, *DRA20*, *DRA19*, *DRA17*, *DRA16*, *DRA2*, *DRA11*, *DRA10*, *DRA12*, *Eqca2*, *Eqca6*, *Eqca7*, and *Eqca8*. Haplotypes *Eqhe* and *DRA9* were separated from *DRA1* by as two mutations step as are most closely related GZP haplotypes. Allele *DRA5* seemed more likely to be ancestral, *Eqbu*, *Eqze*, *Eqgr, Egas* were separated from *DRA5* by one or two mutational step and are most closely related to the GZP haplotypes. Most *DRB* alleles were dispersed throughout the whole network, and there was a closer genetic relationship between GZP and other horse species. Wild ass haplotypes, *Eqas3*, *Eqas4* and *Eqas6,* were separated from *DRB28* by one mutational step, as are most closely-related GZP haplotypes. Furthermore, the haplotypes *DRB2* (*Eqpr1*) and *DRB3* (*Eqpr2*) were shared by GZP and Przewalski’s horse. The haplotypes *DRB1* (*Eqca5*), *DRB2* (*Eqca12*), *DRB4* (*Eqca7*), *DRB5* (*Eqca1*), *DRB15* (*Eqca2*) and *DRB23* (*Eqca8*) were shared between GZP and the European horse.

**Table 4:** Estimation of codon evolution models for the ELA class II *DQA*, *DQB*, *DRA* and *DRB* loci.

Locus	Nested model pairs	p	ln L	Parameter estimates	Site under positive selection
*DQA*	M0: one-ratio	2	−1411.41	*ω* = 1.002	NA
	M3: discrete	6	−1,286.74	*ω*0 = 0.000, p0 = 0.585	NA
				*ω*1 = 0.283, p1 = 0.361	
				*ω*2 = 6.020, p2 = 0.054	
	M1a: nearly neutral	3	−1,336.18	*ω*0 = 0.000, p0 = 0.851	NA
				*ω*1 = 1.000, p1 = 0.149	
	M2a: positive selection	5	−1,281.72	*ω*0 = 0.000, p0 = 0.775	10,17,18,30,46,51,60,61,65,69,70,72
				*ω*1 = 1.000 p1 = 0.205	
				*ω***2** = **8.583**, p2 = 0.020	
	M7: beta	3	−1,338.52	*p* = 0.008, *q* = 0.054	
	M8: beta& *ω*	5	−1,281.73	p0 = 0.979, p1 = 0.021	10,17,18,30,46,51,52,60,61,65,68,69,70,72
				*p* = 0.005, *q* = 0.020, *ω* = **8.425**	
*DQB*	M0: one-ratio	2	−1,682.05	*ω* = 0.449	NA
	M3: discrete	6	−1,461.64	*ω*0 = 0.054, p0 = 0.921	NA
				*ω*1 = 1.283, p1 = 0.073	
				*ω*2 = 6.935, p2 = 0.006	
	M1a: nearly neutral	3	−1,508.29	*ω*0 = 0.025, p0 = 0.951	NA
				*ω*1 = 1.000, p1 = 0.048	
	M2a: positive selection	5	−1,462.34	*ω*0 = 0.043, p0 = 0.913	16,27,57,61
				*ω*1 = 1.000, p1 = 0.081	
				*ω*2 =**6.240**, p2 = 0.005	
	M7: beta	3	−1,519.53	*p* = 0.008, *q* = 0.054	16,27,47,57,61
	M8: beta& *ω*	5	−1,467.39	p0 = 0.994, p1 = 0.005	
				*p* = 0.072, *q* = 0.490, *ω* = **6.373**	
*DRA*	M0: one-ratio	2	−632.76	*ω* = 0.778	NA
	M3: discrete	6	−612.55	*ω*0 = 0.000, p0 = 0.924	NA
				*ω*1 = 0.000, p1 = 0.010	
				*ω*2 = 10.031, p2 = 0.066	
	M1a: nearly neutral	3	−626.11	*ω*0 = 0.000, p0 = 0.687	NA
				*ω*1 = 1.000, p1 = 0.313	
	M2a: positive selection	5	−612.55	*ω*0 = 0.000, p0 = 0.934	12,14,15,16,18,19,49,64,68
				*ω*1 = 1.000 p1 = 0.001	
				***ω*2 = **10.286****, p2 = 0.065	
	M7: beta	3	−627.68	*p* = 0.013, *q* = 0.020	12,14,15,16,18,19,49,64,68
	M8: beta& *ω*	5	−612.54	p0 = 0.934, p1 = 0.065	
				*p* = 0.005, *q* = 6.831, *ω*= 10.323	
*DRB*	M0: one-ratio	2	−1,720.32	*ω* = 0.772	NA
	M3: discrete	6	−1,534.47	*ω*0 = 0.074, p0 = 0.854	NA
				*ω*1 = 1.351, p1 = 0.131	
				*ω*2 = 6.823, p2 = 0.015	
	M1a: nearly neutral	3	−1,580.21	*ω*0 = 0.030, p0 = 0.913	NA
				*ω*1 = 1.000, p1 = 0.087	
	M2a: positive selection	5	−1,535.16	*ω*0 = 0.054, p0 = 0.827	1,2,23,28,48,58,61,62,65,69,77
				*ω*1 = 1.000 p1 = 0.158	
				*ω*2=**5.972**, p2 = 0.015	
	M7: beta	3	−1,583.97	*p* = 0.016, *q* = 0.104	1,2,23,28,47,48,58,61,62,65,69,77
	M8: beta&*ω*	5	−1536.75	p0 = 0.984, p1 = 0.015	
				*p* = 0.087, *q* = 0.328, *ω*=**5.961**	

**Notes.**

p, number of free parameters in the *ω* distribution; ln L, log-likelihood; Model parameter estimates include the nonsynonymous to synonymous rate ratio (*ω*) and proportion of sites (p) under each *ω* site class. Sites under selection were predicted by the Bayes Empirical Bayes (BEB) approach: sites inferred to be under positive selection with posterior probabilities >99%.

## Discussion

Our study revealed the diversity of the four ELA class II gene regions, *DQA*, *DQB*, *DRA* and *DRB*, and the contribution of many novel alleles identified in GZP. Our data determined within-species variation using the numbers of alleles. 21 *DQA* alleles, 45 *DQB* alleles, 22 *DRA* alleles, and 31 *DRB* alleles were unequivocally identified from the GZP.

The *DRA* locus was relatively well-conserved in four GZP loci compared with the other three loci. The alignments of the *DQA*, *DQB*, and *DRB* genes revealed considerable sequence diversity. However, *DRA* had a lower nucleotide diversity. Our results are consistent with the level of nucleotide diversity at the genus level for Equus *ELA* genes as reported by Kamath & Getz (2011). *DQB* had the highest level of polymorphisms with a ratio of polymorphic sites of 46.08%, this was followed by *DRB* and *DQA*. *DRA* had the lowest level of polymorphisms (15.04%), which was consistent with the results of the *DRA* locus in dogs (Wagner, Burnett & Storb, 1999), cats (Yuhki & O’Brien, 1997), goats (Takada et al., 1998), and pigs (Chardon, Renard & Vaiman, 1999). The genetic diversity of ELA is reportedly important for immune functions involving the resistance and susceptibility to pathogens (Trowsdale & Parham, 2004) with a probable mechanism of gene selection in the evolution process of the pony (Penn & Potts, 1999).

We detected the balancing selection events by determining the rate of non-synonymous/ synonymous substitutions (*d*_N_/*d*_S_ ratio) of nucleotides. Our results revealed a high genetic variability at the *DQA*, *DQB*, *DRA*, and *DRB* loci. The *d*_N_/ *d*_S_ ratio (*d*_N_/ *d*_S_ = 0.560) at the *DRA* locus was the lowest, which was similar to the low levels of polymorphisms detected by sequence alignment. It has been established that the number of synonymous substitutions is greater than non-synonymous substitutions due to strong functional and structural constraints on the protein (Kamath & Getz, 2011). The number of polymorphisms at the *DRA* locus may be attributed to the selective pressure for DRA haplotypes that present pathogenic antigens for the host species more efficiently (Albright-Fraser et al., 1996).

We found nine *DQA* codons, five *DQB* codons, nine *DRA* codons, and seven *DRB* codons under significant positive selection. The majority of these codons were predicted to be the ABS of *ELA*. The amino acids under site-specific selection were located on the protein surface based on SWISS-MODEL prediction results (Fig. 4) and were found on the inner surface of the MHC cleft with bound peptides in the antigen presentation (Madden et al., 1995). Several reports indicated that the diversity and nonsynonymous mutations at the ABS could improve the hosts ability to recognize pathogens (Hughes & Nei, 1988; Hughes & Nei, 1989). These data suggest that the different rates of non-synonymous and synonymous substitutions in *DQA, DQB, DRA* and *DRB* were closely related to the ABS changes in the GZP. In particular, the *d*_N_/ *d*_S_ ratio in the ABS was greater than that in the non-ABS region at the *DQA, DQB* and *DRB* loci, which is common in the Argentine Creole horse (Díaz et al., 2001). The *d*_N_/ *d*_S_ ratio of ABS was higher than the other regions, which may be due to balancing selection (Albright-Fraser et al., 1996), and the positive selection results in MHC polymorphisms (Yang et al., 2000).

The haplotype median network of *DQA*, *DQB*, *DRA* and *DRB* between GZP and other horses (*Eqca*, *E. callabus*; *Eqpr*, *E. przewalski*; *Eqki*, *E. kiang*; *Eqgr*, *E. grevyi*; *Eqas*, *E. asinus*; *Eqbu*, *E. burchelli*; *Eqze*, *E. zebra*; *Eqhe*, *E. hemionus*) were analyzed. Among these, several wild ass haplotypes were separated from *DQA1*, *DQB1*, and *DRB28* by one or two mutational steps and are more closely related to GZP haplotypes. The divergence time between the horse and ass has been estimated to be 0.88–2.3 Ma (Krüger et al., 2005). One *E. hemionus* haplotype (*Eqhe2*) was separated from *DRA* by two mutational steps and is most closely related to GZP haplotypes. It suggested that *DQA1*, *DQA3*, *DQB1*, *DRA1*, *DRA5*, and *DRB28* may be the oldest alleles. The haplotypes *DRB2* and *DRB3* were shared between GZP and the Przewalski’s horse at the *DRB* locus. Przewalski’s horse haplotypes *Eqpr3*, *Eqpr4* and *Eqpr2* were separated from *DQA3* by two mutational steps. Przewalski’s horse was discovered on the Asian steppes in the 1870s and it is the only surviving species of wild horse in the world (Wakefield et al., 2002). It is thought that the Przewalski’s horse and the domesticated horse populations separated about 45,000 years ago and maintained a certain degree of gene-flow (Der Sarkissian et al., 2015). Some haplotypes were shared between the GZP and European horses, including *DQA1*, *DQA3*, *DQB5*, *DQB13*, *DRB1*, *DRB2*, *DRB15*, *DRB23*, *DRB4*, and *DRB5*. The allele *DQA1* appears to be the ancestor for the three alleles, *Eqca10*, *Eqca20*, and *Eqca19*. Allele *DRA1* seemed more likely to be ancestral for four alleles, including *Eqca2*, *Eqca6*, *Eqca7,* and *Eqca8*. The genes of the domesticated Asian horse may have dispersed into European populations because of the gene flow (Bjørnstad, Nilsen & Røed, 2003). Interestingly, the haplotypes DQA1, DQA3, and DRA5 were shared between the GZP and E.burchelli, E.grevyi and zebra. The divergence time between horses and zebras is estimated to be 0.86–2.3 Ma based on microsatellite trees (Krüger et al., 2005). The common ancestor of all extant forms may have existed about 3.9 Ma, and speciation leading to the zebra, ass, and horse may have occurred within the following 0.5 Ma (George & Ryder, 1986). These data and our results indicated that the GZP is an ancient variety of equid. Additional studies on the GZP may advance our knowledge of unique haplotypes and their roles in the adaptation to local environmental pressures such as the unique pathogenic microorganisms in the mountainous and humid districts in Guizhou province, China.

## Conclusion

Nucleotide diversity was detected from exon 2 of *ELA-DQA*, *DQB*, *DRA*, and *DRB* genes in the GZP using direct sequencing technology. Of those four loci, the *DRA* locus was relatively well-conserved and possessed the lowest diversity. Many codons in the ABS underwent positive selection, including nine *DQA* codons, five *DQB* codons, nine *DRA* codons, and seven *DRB* codons. The amino acids coded by selected codons were found on the inner surface of the cleft of the *ELA* complex and were bound to an antigen peptide. The selected sites may be related to the GZP’s ability to defend against foreign pathogens from the surrounding habitat. Many ancient alleles were detected at the *DQA*, *DQB*, *DRA* and *DQB* gene regions of GZP. Two older haplotypes of *DRB* (*DRB2* and *DRB3*) were shared by the GZP and Przewalski’s horse. Two older haplotypes of *DRA* (*DRA1* and *DRA5*) were separated from *Eqbu*, *Eqze*, *Eqgr*, and *Egas* by one or two mutations steps, and four older haplotypes of GZP (*DQA1*, *DQA3*, *DQB1*, and *DRB28*) were closer to the wild ass and Przewalski’s horse by only one or two mutational steps. The indigenous breed, GZP, may have retained ancient haplotyes in *ELA* genes. There may be a large number of unique haplotypes dispersed in GZP resulting from the long process of ELA molecule evolution. The unique genetic characteristics of GZP have been unclear, undervalued, and confused with other ponies. The genetic uniqueness revealed in our study is helpful to understand its genetic conservation of this ancient variety of pony.

##  Supplemental Information

10.7717/peerj.9889/supp-1 Supplemental Information 1 The nucleotide of the DQA, DQB, DRA, and DRB locus

10.7717/peerj.9889/supp-2 Supplemental Information 2 Nucleotide composition of DQA, DQB, DRA, and DRB in Guizhou pony

## Additional Information and Declarations

### Data Availability

The following information was supplied regarding data availability: Raw data is available as Supplemental Files.
